# Segregostat: a novel concept to control phenotypic diversification dynamics on the example of Gram‐negative bacteria

**DOI:** 10.1111/1751-7915.13442

**Published:** 2019-05-29

**Authors:** Hosni Sassi, Thai Minh Nguyen, Samuel Telek, Guillermo Gosset, Alexander Grünberger, Frank Delvigne

**Affiliations:** ^1^ Terra Research and Teaching Centre Microbial Processes and Interactions (MiPI) Gembloux Agro‐Bio Tech University of Liège Gembloux Belgium; ^2^ Departamento de Ingeniería Celular y Biocatálisis Instituto de Biotecnología Universidad Nacional Autónoma de México Cuernavaca, Morelos México; ^3^ Multiscale Bioengineering Bielefeld University Universitätsstraße 25 33615 Bielefeld Germany

## Abstract

Controlling and managing the degree of phenotypic diversification of microbial populations is a challenging task. This task not only requires detailed knowledge regarding diversification mechanisms but also advanced technical set‐ups for the real‐time analyses and control of population behaviour on single‐cell level. In this work, set‐up, design and operation of the so called segregostat are described which, in contrast to a traditional chemostat, allows the control of phenotypic diversification of microbial populations over time. Two exemplary case studies will be discussed, i.e. phenotypic diversification dynamics of *Eschericia coli* and *Pseudomonas putida* based on outer membrane permeabilization, emphasizing the applicability and versatility of the proposed approach. Upon nutrient limitation, cell population tends to diversify into several subpopulations exhibiting distinct phenotypic features (non‐permeabilized and permeabilized cells). Online analysis leads to the determination of the ratio between cells in these two states, which in turn triggers the addition of glucose pulses in order to maintain a predefined diversification ratio. These results prove that phenotypic diversification can be controlled by means of defined pulse‐frequency modulation within continuously running bioreactor set‐ups. This lays the foundation for systematic studies, not only of phenotypic diversification but also for all processes where dynamics single‐cell approaches are required, such as synthetic co‐culture processes.

## Introduction

Controlling and managing the degree of phenotypic diversification of microbial populations has recently attracted a lot of attention (Ackermann and Schreiber, 2015; Binder *et al*., 2017), due to our expanding knowledge about noise of biological systems (Eldar and Elowitz, 2010). This trend is promoted by the emergence of new disciplines, such as cybergenetics (Milias‐Argeitis *et al*., 2016; Benzinger and Khammash, 2018), applying control theory for managing cell‐to‐cell heterogeneity in gene expression at a very high spatio‐temporal resolution. However, these studies have mostly been conducted in microfluidic cultivation devices. A more conventional device that is thoroughly used for microbial physiology studies is the chemostat, allowing the long‐term cultivation of microbial population in defined limiting conditions (Wides and Milo). The main reason behind the intense use of this cultivation device is because it was recognized as being able to stabilize microbial population in a given physiological state. However, results accumulated in recent years pointed out that this is not true (Wides and Milo), and the impact of phenotypic diversification has not been taken into account so far. Indeed, chemostat cultivations lead to a very competitive environment generating many successive takeovers of different subpopulations that are non‐detectable at the biomass or substrate levels. Therefore, novel tools need to be established based on combination of single‐cell technologies, such as flow cytometry (FC), and process engineering approaches.

**Figure 1:**
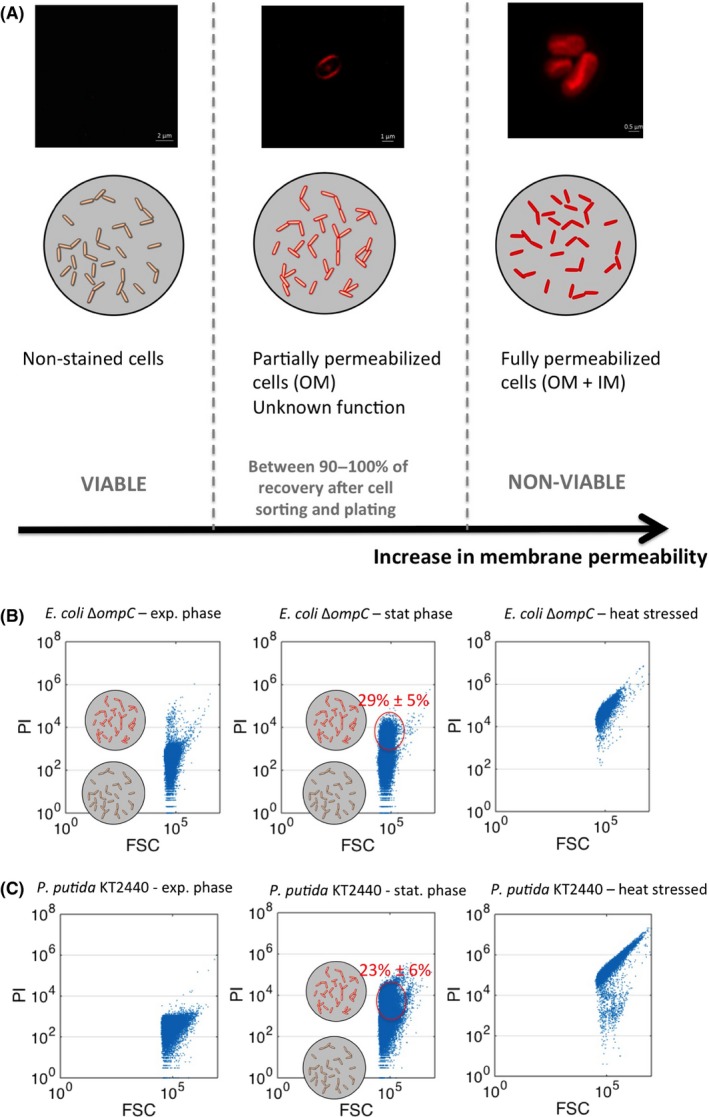
A. With the three physiological state according to PI staining (each state is illustrated by images acquired by high resolution microscopy (confocal microscopy with Airyscan detector). B. Flow cytometry experiments (*x*‐axis corresponding to forward scatter or FSC;* y*‐axis corresponding to red fluorescence related to PI staining) with *E. coli* taken at different cultivation stages (cultures made in flasks). C. Flow cytometry experiments (*x*‐axis corresponding to forward scatter or FSC;* y*‐axis corresponding to red fluorescence related to PI staining) with *P. putida* taken at different cultivation stages (cultures made in flasks).

Nutrient stress is a strong trigger of phenotypic diversification that can be eventually used for controlling the degree of diversification of a bacterial population. Previous studies have shown that such phenotypic diversification mechanisms are governed by a complex set of physiological mechanisms involving noise in gene expression, metabolism and growth (Kiviet *et al*., 2014; Kleijn *et al*., 2018; Patange *et al*., 2018), these three mechanisms being highly cross‐correlated and being controlled through mutual feedback loops. Ultimately, the superimposition of these three mechanisms leads to population phenotypic heterogeneity that can in turn confer interesting functionalities to the whole population (e.g. bet‐hedging, division of labour) (Ackermann, 2015). Most of the works focused on phenotypic diversification of microbial populations have been carried out based on single‐cell proxies involving either GFP expression (used as a proxy for noise in gene expression) (Nikolic *et al*., 2013; Baert *et al*., 2015) or growth rate (Dusny *et al*., 2012; Grunberger *et al*., 2013). In the context of this work, we propose to focus on another relevant single‐cell proxy, i.e. outer membrane (OM) permeabilization. Indeed, membrane permeability is a fundamental physiological parameter driving the way that microbes respond to environmental cues and, eventually, adapt to stresses (Ferenci, 1996). However, like growth rate (van Heerden *et al*., 2017), membrane permeability is a physiological process involving an intricate set of genes and regulation processes. It is thus of importance to develop advanced single‐cell technologies for characterizing such physiological parameter. This phenomenon is depicted in Fig. 1 for *Escherichia coli* BW25113 Δ*ompC* and *Pseudomonas putida* KT2440. Propidium iodide (PI) is the most frequently used fluorescence indicator for cell viability based on assessment of membrane integrity (Shi *et al*., 2007). PI is also a very stable molecular probe that can be used in combination with online FC to get an instantaneous snapshot of the physiological status of cells, by comparison with GFP‐based biosensors that are typically exhibiting lag time due to transcriptional‐ and translational‐dependent dynamics for the activation/degradation of the fluorescent protein (Delvigne *et al*., 2015). The dynamics of outer membrane (OM) permeabilization will be considered as a model system for dynamic high‐throughput single‐cell analyses. More precisely, population profiling by online FC will be performed in chemostat mode. Based on this information, subpopulation ratio will be controlled based on a feedback control loop working based on FC data in a device we called segregostat. This device is able to trigger glucose pulses at given time interval, based on the ratio between the non‐permeabilized and OM‐permeabilized subpopulations. These glucose pulses generate alternating conditions between nutrient excess and nutrient limitation, further modulating the appearance of OM‐permeabilized cells.

## Results: Membrane permeabilization dynamics in *E. coli* and *P. putida* upon nutrient limitation

One of the critical steps for studying the outcome of phenotypic diversification relies on the identification of relevant single‐cell proxies. In this work, we have identified propidium iodide (PI) as an effective biomarker for cells switching to adaptation in nutrient limitation (Fig. 1). PI will be used for keeping track of the progressive permeabilization of the OM upon nutrient limitation. Indeed, we have observed that, during switch from exponential phase to stationary phase in flask culture experiments, Gram‐negative bacteria undergo OM permeabilization, leading to an intermediate PI‐stained fraction of cells (Fig. 1B for *E. coli* and Fig. 1C for *P. putida*). Additionally, these OM‐permeabilized cells exhibit different metabolic features as observed during dual‐staining experiments (see Appendix S2). For the two Gram‐negative bacteria, OM‐permeabilized cells exhibited fluorescence associated with RedoxSensor Green (RSG) and 2‐NBDG uptake, suggesting that these cells are metabolically active. However, differences have been observed at the level of fluorescence intensity. For *E. coli*, dual‐staining experiments revealed that OM‐permeabilized subpopulation exhibited reduced glucose uptake, as indicated by 2‐NBDG staining, and enhanced electron transport chain, as indicated by RSG staining. For *P. putida*, the opposite trend has been observed, i.e. OM‐permeabilized cells exhibited slightly reduced electron transport activity based on RSG staining and slightly enhanced glucose uptake capacity based on 2‐NBDG. These data suggest that the physiological mechanisms behind OM permeabilization are triggered by different mechanisms in *E. coli* and *P. putida*. These potential mechanisms will be developed in the discussion section.

**Figure 2:**
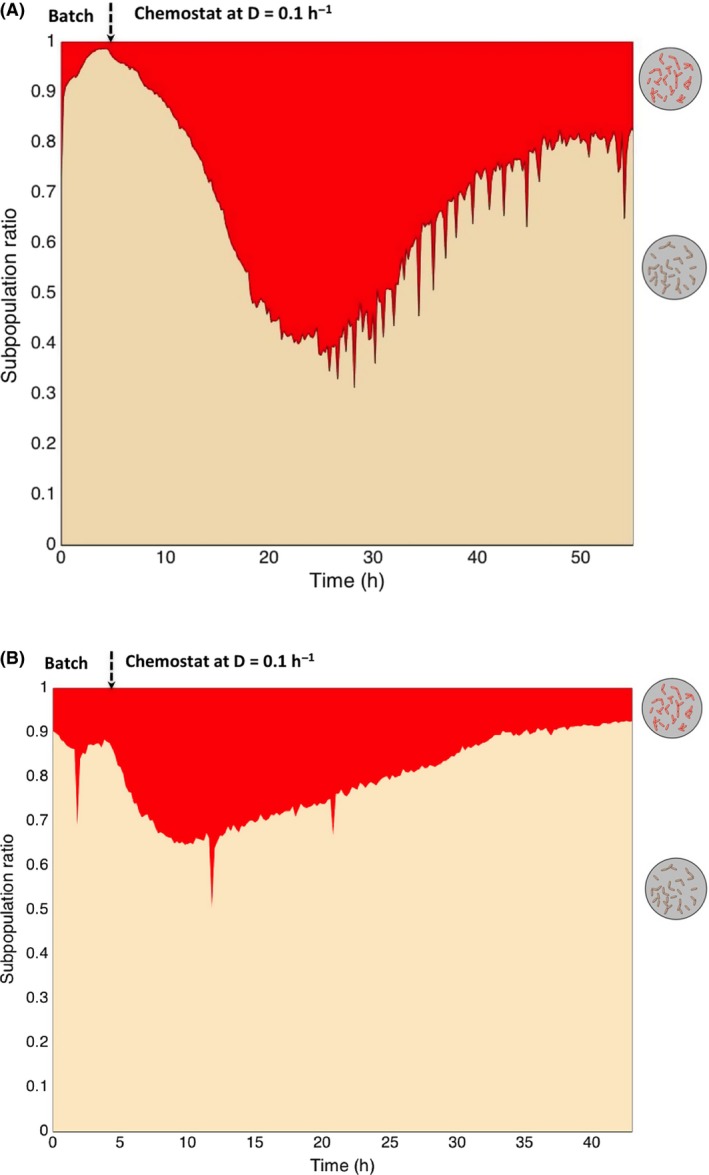
Dynamics of phenotypic diversification in chemostat for A: *E. coli*; B: *P. putida*.

Since this particular phenotype seems to be triggered by nutrient limitation, chemostat experiments at low dilution rate, i.e. D = 0.1 h^−1^, were carried out. Indeed, it has been previously observed that cultivating *E. coli* at this dilution rate triggers adaptation to nutrient limitation, notably through a complete remodelling of the porins at the level of the OM (Liu, 1998). It has also been shown that this dilution rate is in the range of growth rate for which active porin remodelling takes place (Ferenci, 2001). On this basis, chemostat experiments were performed both for *E. coli* and *P. putida* with online FC profiling (Fig. 2). Similar trends have been observed for *E. coli* and *P. putida*. In the first phase with increasing OM‐permeabilized subpopulation, followed by a second phase where this subpopulation decreases. Other interesting observation for each species is a continuous evolution at the level of their subpopulation ratio, whereas a chemostat is typically used for ‘stabilizing’ microbial population (Wortel *et al*., 2016; Wides and Milo). In front of the results, *E. coli* exhibits a lower phenotypic diversification rate than *P. putida*. Indeed, if we compute the rate of diversification from the first phase of a chemostat where the OM‐permeabilized subpopulation increases, the value is 0.044 h^−1^ for *E. coli* against 0.085 h^−1^ for *P. putida*. These experiments show that it is not possible to maintain cell population heterogeneity within standard chemostat experiments. Indeed, the subpopulation dynamics that have been observed lead to the conclusion that data obtained based on chemostat cultivation might need to be reanalysed in the light of the presence of phenotypically different subpopulations.

**Figure 3:**
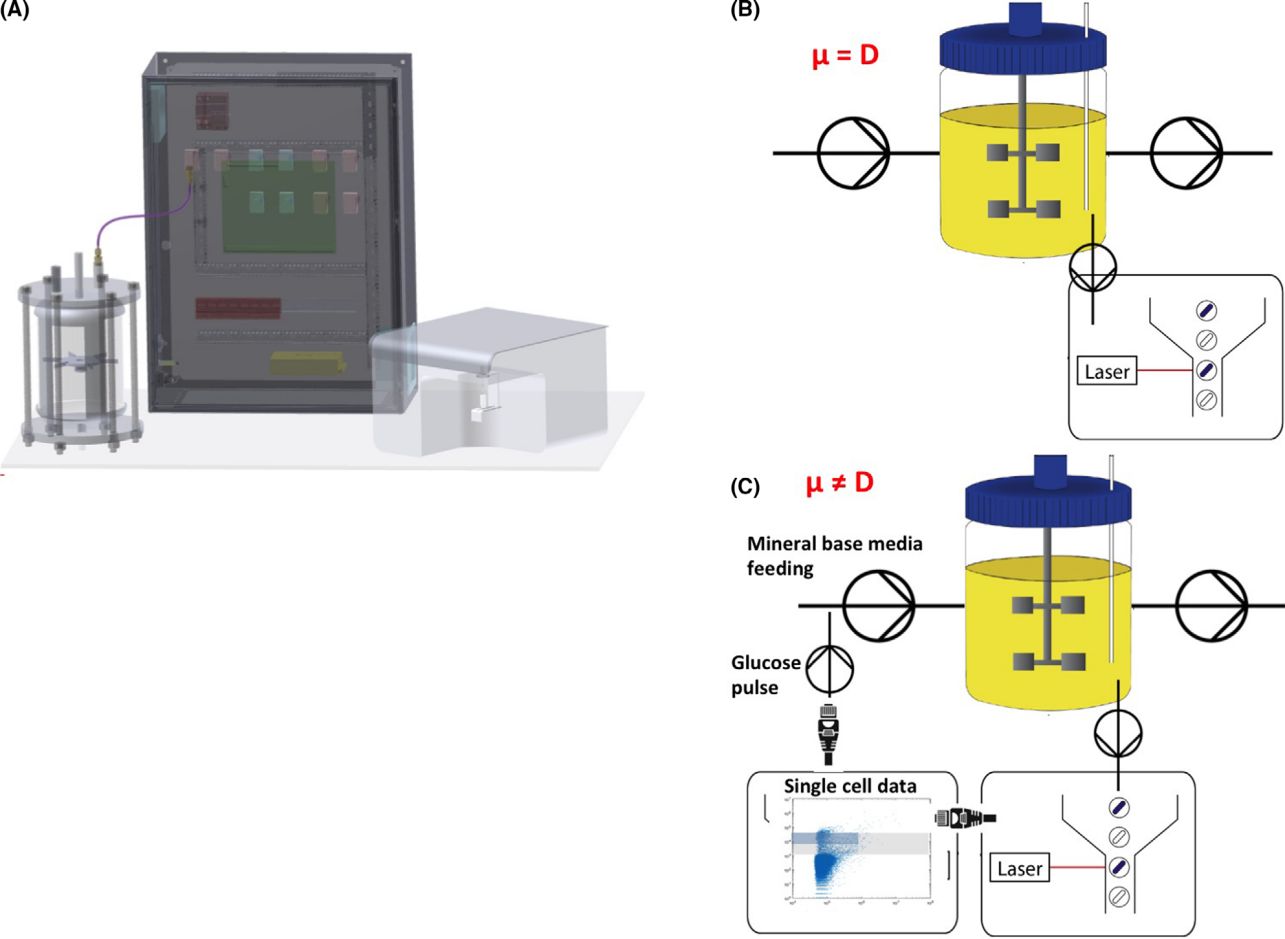
A. Scheme with the different components for the online FC platform. B. Use of the platform in the chemostat mode. C. Use of the platform in the segregostat mode. It is important to keep in mind that during segregostat, glucose pulses are added based on population diversification, and therefore, the growth rate (μ) is not necessarily equal to the dilution rate (D).

**Figure 4:**
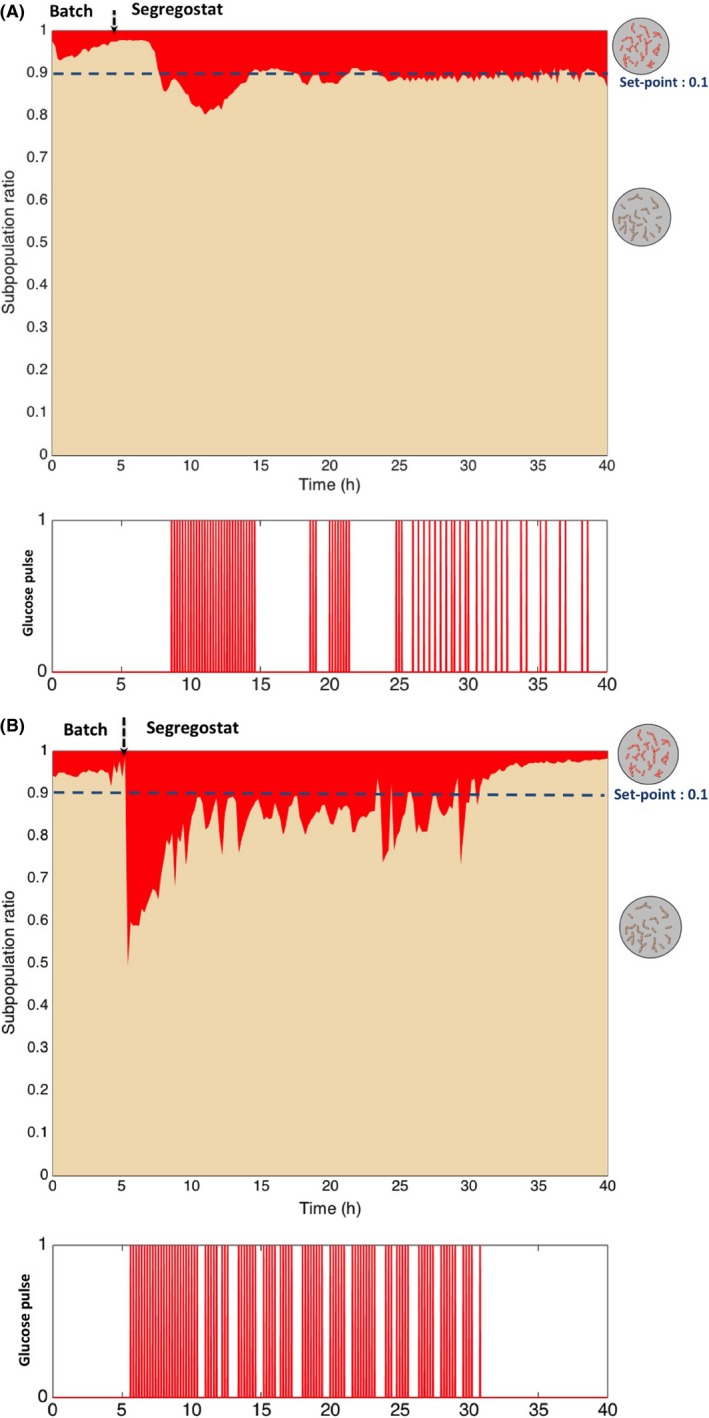
Dynamics and control of phenotypic diversification in segregostat for (A) *E. coli*; (B) *P. putida*. Glucose pulses have been made based on a population diversification ratio of 0.1.

Based on these data, we designed an alternative cultivation device for maintaining cell population heterogeneity (or cell subpopulation ratio) at a constant level with time. This device, which we called segregostat (see Fig. 3 for a description of the set‐up), is also running in continuous mode, but is under the control of the degree of diversification of the microbial population, i.e. in our case the ratio between cells in non‐permeabilized and OM‐permeabilized subpopulation. Continuous monitoring of cell population is ensured by online FC like in chemostat experiments. However, in the case of the segregostat, the system is continuously fed with base medium without any carbon source. Glucose is pulsed at given time interval based on the outcome of flow cytometry analysis. Pulses have been performed in order to avoid glucose accumulation during the cultivation (dissolved oxygen profiles are provided in Appendix S3). Since the switch of cells to the permeabilized state is triggered by carbon limitation, glucose pulse can be used as an efficient actuator for limiting this phenotypic switch. During these experiments, glucose pulse is triggered automatically when the amount of cells in the permeabilized subpopulation exceed 10% of the total amount of cells (displayed as a subpopulation ratio of 0.1 on Fig. 4). This threshold has been considered here as being a significant proxies for the induction of the diversification process.

The *E. coli* and *P. putida* profiles displayed clear similitudes, but also some differences. First, upon the entry in the segregostat mode, both Gram‐negative bacteria exhibited a strong phenotypic diversification traduced by a series of glucose pulses (Fig. 4; ~5–10 h). However, this process seems to be faster for *P. putida* than for *E. coli*. Indeed, a nice feature of the segregostat is that the diversification periods can be easily tracked based on the glucose pulse profile. Indeed, these Gram‐negative bacteria tend to induce OM permeabilization upon nutrient limitation, this process is being controlled by glucose pulses. Then, a phase with successive glucose pulses corresponds to a period of intense phenotypic diversification. Diversification process is faster for *P. putida*, with an average 3.9 pulses per h, in comparison with *E. coli* for which the average is 2.28 pulses per h. These observations are in good accordance with the results gained through chemostat experiments pointing out that the diversification rate is approximately twice as much for *P. putida* than for *E. coli*.

**Figure 5:**
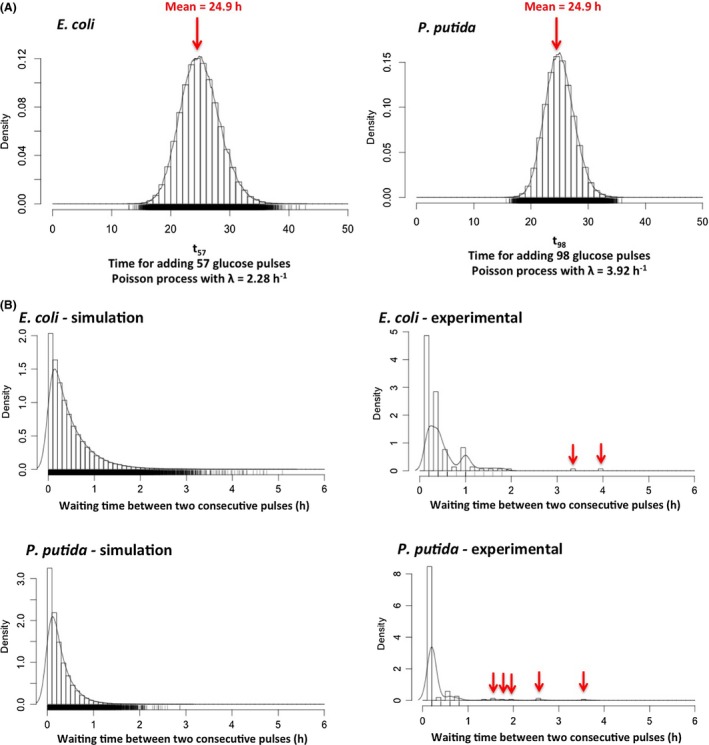
Analysis of glucose pulses addition profiles in segregostat. A. Total time needed for adding 57 and 98 glucose pulses, respectively, according to a theoretical Poisson process (the number of pulses, as well as the rate λ of the Poisson process, has been determined from the experimental segregostat data). B. Distribution of waiting time between two consecutive glucose pulses according to a theoretical Poisson law (simulation) and from the segregostat data (experimental).

At this level, it was investigated whether the pulses addition follow a stochastic trend. For this purpose, stochastic simulations have been performed based the rate of glucose pulse addition previously determined through the segregostat experiments (here considered as the transition rates (λ, in h^−1^) for stochastic simulations). Poisson processes were simulated based on the average number of pulses per hours and the total pulses injected over the segregostat cultures for the two Gram‐negative models (Fig. 5A). Interestingly, it can be observed that the mean time computed from the distribution, i.e. 24.9 h, matches very well with the experimental time which is roughly around 25 h.

However, when looking at the glucose pulse profiles displayed in Fig. 4, it can be observed that we are diverging from a classical Poisson process. It is particularly obvious in the case of *P. putida* where bursts in glucose pulses are observed, i.e. a period with successive pulses followed by period with no pulses. In order to highlight this phenomenon, we computed the waiting time distribution, i.e. in our case, this is described by the time between two successive glucose pulses. Stochastic simulations involving Poisson processes have been run with the transition rate determined experimentally, and the results have been compared with the distribution acquired experimentally (Fig. 5B). There are two interesting features that need explanation. The first feature relies on the fact that, in the experimental distribution, a lot of low waiting time values are non‐represented, whereas some others are overrepresented. This can be explained that in the segregostat set‐up, glucose pulses are added in discrete time intervals (glucose pulse is potentially injected every 12 min depending on the degree of diversification of the microbial population), whereas stochastic simulations are carried out in continuous time. The second, and most interesting value, is that some high waiting time values, not predicted by stochastic simulations, can be noticed in both cases, but the effect is more obvious for *P. putida* (these values has been pointed out by red arrows for the experimental distribution displayed in Fig. 5). These values correspond to the arrests in glucose pulses profiles displayed in Fig. 4 and are typical of a bursting process, i.e. process exhibiting period of intense diversification, followed by periods with no activities. Possible explanations about the source of such burst behaviour will be discussed in the next section.

## Discussion

Phenotypic diversification is due to stochasticity in gene expression, arising from fluctuations in transcription and translation despite constant environmental conditions. These mechanisms have been the focus of intensive researches during the last decade and have led to a coherent mathematical and experimental framework of molecular stochasticity in prokaryotic and eukaryotic systems (Kaern *et al*., 2005; Eldar and Elowitz, 2010). This framework has been notably used in order to decipher the impact of regulatory network structure on the propagation (Blake *et al*., 2003; Kleijn *et al*., 2018) and control of phenotypic diversification (Milias‐Argeitis *et al*., 2016; Briat and Khammash, 2018; Briat *et al*., 2018), as well as on the possible functionality of such diversification (Eldar and Elowitz, 2010; Levine *et al*., 2013; Ackermann, 2015). However, most of these researches have been conducted at low spatio‐temporal resolution, i.e. either on a limited numbers of cells, or focused on given time point. In this work, we have developed a tool, called segregostat, allowing to expand the methodology to a very high amount of cells with a high temporal resolution. Indeed, the segregostat allowed generating high‐quality subpopulation data. Such data are rarely available at such population density and should help paving the way for further characterization of microbial cell population diversification dynamics. One characteristic feature of the version of the segregostat that has been shown in this work is that population control is made based on glucose pulses. The resulting glucose pulse profile can then be used for identifying the period of time for which intense phenotypic diversification occurs. Interestingly, it has been shown that the time between two consecutive glucose pulses was not following a simple Poisson process, but was rather occurring in a burst fashion. Transcriptional and translational bursting are known to occur at the single‐cell level and drive intrinsic noise leading to phenotypic diversification (Kaern *et al*., 2005; Eldar and Elowitz, 2010). However, this process has never been described for a whole microbial population and an outstanding question would be to understand how such typical single‐cell dynamics can be transposed to the behaviour of whole microbial population. One hypothesis is that glucose pulsing results in synchronization of microbial subpopulations dynamics during cultivation in chemostat. Indeed, since control is made based on glucose pulsing, the microbial population is exposed to cycles of nutrient excess and limitation, based on a given diversification state. This hypothesis needs of course confirmation and extra work is still required in order to fully understand the molecular mechanisms behind the appearance of OM‐permeabilized subpopulations, but the dynamic data acquired in this work allow to point out some observations. First, by coupling PI staining with either 2‐NBDG and RSG staining, different observations were made for *E. coli* and *P. putida*. Indeed, for *E. coli*, the OM‐permeabilized subpopulation exhibits different metabolic features than non‐permeabilized cells, i.e. a decreased glucose uptake capacity and an increased electron transport chain (Appendix S2). In the case of *P. putida*, the opposite trend was observed. This could be explained by the fact that, from a mechanistic point of view, glucose uptake mechanisms and catabolism reactions between *E. coli* and *P. putida* are very distinct. These differences could in part be responsible for the observed dynamics. In *E. coli,* all the glycolytic reactions take place in the cytoplasm. In contrast, *P. putida* carries out some of the reactions leading to glucose catabolism in the periplasm. Indeed, it has been shown that small amounts of gluconate and 2‐ketogluconate accumulate in the extracellular space when *P. putida* grows on glucose (del Castillo *et al*., 2007). These metabolic aspects will be developed further in this section. For *E. coli*, a simple glucose pulsing strategy can be used for controlling phenotypic diversification rate and maintaining microbial population in a given degree of heterogeneity with time. For *P. putida*, glucose pulsing leads to oscillations around the subpopulation ratio that has been used as the set point for the pulse addition, and eventually leads to a loss of control at the end of the cultivation. It seems thus that, in this case, an advanced control strategy based on predictive model will be required.

Taken altogether, the data gathered in this work point out that the OM‐permeabilization mechanism has different origins in *E. coli* and *P. putida*. Ordinarily, PI molecule carry two positive charges, one of which seems open to the surroundings and should prevent its membrane permeation. In a few conditions, such as high ATP turnover and nutrient limitation, a lower membrane potential will amplify the ion‐motive force for cations, particularly if they carry two charges such as propidium iodide (Bot and Prodan, 2010). Consequently, a decreased membrane potential might facilitate diffusion of PI molecules inside the periplasmic space. Although different causes seem to be at the basis of PI diffusion through the OM of the two Gram‐negative bacteria investigated, its dynamics can be easily tracked by the online FC platform. However, if some underlying physiological mechanisms can be advanced for *E. coli*, this physiological process has not been reported for *P. putida*. The outer membrane of Gram‐negative bacteria is composed of an asymmetric lipid bilayer, phospholipids (PLs) being located in the inner leaflet of the membrane and lipopolysaccharides (LPS) being located to the outer leaflet. In *E. coli*, the MlaA wall surrounding the channel impairs transposition of PLs from inner to outer leaflet. By contrast, outer leaflet PLs can enter this channel and are removed from the OM via transfer to MlaC with the particularly interaction of protein membrane (Chong *et al*., 2015; Yeow *et al*., 2018). The combined function of porin OmpC and MlaC in lipid transport and maintenance of membrane asymmetry has been observed (Chong *et al*., 2015). In addition to the disruption in OM lipid asymmetry, cells lacking OmpC exhibit OM permeability defects, including increased sensitivity to SDS/EDTA and detergents. The authors also suggest that OM permeability defects in Δ*ompC* cells are not simply a result of disruption in OM lipid asymmetry and OmpC may be important for other processes that influence the integrity and function of the OM. Membrane asymmetry is also known to impact different bilayer properties, including cell shape, surface charge, permeability and membrane potential (Marquardt *et al*., 2015). Hence, removing OmpC causes imbalance in OM lipids components that affect cell morphology, membrane integrity and membrane potential. Such increase in permeability for Δ*ompC* mutant has also been observed in this work (Appendix S1). However, this mechanism seems to be subjected to high cell‐to‐cell heterogeneity since two clearly defined subpopulations can be observed upon PI staining (Fig. 1B). Porins’ organization and regulation have been less characterized in the case of *P. putida*. By analogy with *E. coli*, these porins are organized in different clusters involving membrane stabilization, cell structure determination, transport of specific substrates and pore formation (Hancock and Brinkman, 2002). Among them, OprB has been more thoroughly investigated and present a high homology with OprB from *P. aeruginosa* and has been suggested to be involved in glucose uptake. In *E. coli*, upon its entry into the periplasm, glucose is phosphorylated by the phosphoenolpyruvate:sugar phosphotransferase system. Catabolism of the generated glucose‐6‐phosphate proceeds by the glycolytic pathway (Gosset, 2005). In contrast, *P. putida* has an incomplete glycolytic pathway since it lacks 6‐phosphofructokinase (Nikel *et al*., 2014). Therefore, it metabolizes glucose via the Entner–Doudoroff pathway, where 6‐phosphogluconate is the key intermediate. When glucose enters the periplasm in this organism, it can be imported to the cytoplasm via an ABC transporter and then phosphorylated by glucokinase. Glucose is also substrate of the periplasmic enzymes glucose dehydrogenase and gluconate dehydrogenase, yielding gluconate and 2‐ketogluconate respectively. These two compounds and glucose‐6‐P are the substrates of three convergent pathways leading to the synthesis of 6‐phosphogluconate (del Castillo *et al*., 2007). These structural differences at the level of glucose uptake between the two organisms can explain the differences observed at the level of 2‐NBDG uptake (Appendix S2).).

Porin organization and regulation, as well as metabolic pathways regulation, are fundamentally different between *E. coli* and *P. putida*. However, these microbes display similar features in terms of phenotypic diversification upon nutrient limitation. Indeed, PI staining reveals in both cases two clearly defined subpopulations, i.e. the first one exhibiting no PI uptake and the second one exhibiting partial staining probably due to the localization of PI molecules inside the periplasm upon OM permeabilization (Fig. 1).

The methodology presented in this work, based on the use of the segregostat, will be an important component of the experimental and numerical workflow needed for addressing these very complex physiological processes. This work points out the importance of choosing an appropriate single‐cell proxy for controlling phenotypic diversification and that the resulting online profiling of subpopulations can lead to a more thorough understanding of population dynamics.

In conclusion, the segregostat can in future be used for several potential applications ranging from: generating population of constant structure over time to be used for extended physiological studies, controlling bioprocesses and ‘homogenizing’ bioprocess populations, analysing and controlling synthetic co‐culture processes, and at a global level, expand our knowledge about the dynamics of phenotypic diversification of microbial populations with a possible link to the functionality of this diversification process.

## Experimental procedures

### Strains and medium composition

The strains used in this study are *E. coli* JW2203‐1 Δ*ompC* (obtained from the Keio collection (Baba *et al*., 2006). Genotype :Δ*lacZ*4787(::*rrnB*‐3), λ^‐^, Δ*ompC*768::kan, rph‐1, Δ(*rhaD‐rhaB*)568, hsdR514) and *Pseudomonas putida* KT2440 (kindly provided by Prof. Pablo I. Nikel, Denmark Technical University, Lyngby). *Escherichia coli* JW2203‐1 Δ*ompC* have been selected based on a prescreening test since it was able to display a higher diversification ratio by comparison with wild‐type and other porin mutants (see Appendix S1). All strains are maintained at −80°C in working seed vials (2 mL) in solution with LB medium and with 30% of glycerol (w/v). Precultures and cultures have been performed on a defined mineral salt medium containing (in g l^−1^): K_2_HPO_4_ 14.6, NaH_2_PO_4_.2H_2_O 3.6; Na_2_SO_4_ 2; (NH_4_)_2_SO_4_ 2.47, NH_4_Cl 0.5, (NH_4_)_2_‐H‐citrate 1, glucose 5, thiamine 0.01, antibiotic 0.1. Thiamine is sterilized by filtration (0.2 μm). The medium is supplemented with 3 ml l^−1^ of trace element solution, 3 ml l^−1^ of a FeCl_3_.6H_2_O solution (16.7 g l^−1^), 3 ml l^−1^ of an EDTA solution (20.1 g l^−1^) and 2 ml l^−1^ of a MgSO_4_ solution (120 g l^−1^). The trace element solution contains (in g l^−1^): CoCl_2_.H_2_O 0.74, ZnSO_4_.7H_2_O 0.18, MnSO_4_.H_2_O 0.1, CuSO_4_.5H_2_O, CoSO_4_.7H_2_O. The medium was supplemented with 5 g l^−1^ of glucose, and antibiotic (kanamycin 25 μg ml^−1^) was added for the cultivation of the *E. coli* JW2203‐1 Δ*ompC* strain.

### Chemostat cultivations

Bioreactor experiments were performed from overnight precultures performed in 1 l baffled flasks containing 100 ml of culture medium and stirred with 200 rpm at 37°C. Cultures in chemostat and segregostat mode were performed in lab‐scale stirred bioreactor ((Biostat B‐Twin, Sartorius) total volume: 2 l; working volume: 1 l). For the batch phase, the overnight cultures were diluted into 1 L of minimal medium at an initial OD_600_ of 0.5. The pH was maintained at 6.9 by automatic addition of ammonia or phosphoric acid. The temperature was maintained at 37°C under continuous stirring rate of 800 rpm and aeration rate of 1 VVM. Upon glucose depletion, observed typically after 4–6 h with a sudden increase in dissolved oxygen, the chemostat or segregostat (see next section) mode is started.

For chemostat cultivations, the medium was continuously fed with the complete minimal medium at a dilution rate of 0.1 h^−1^.

### Online flow cytometry platform

The platform employed is an improved version of a previous online FC platform (Brognaux *et al*., 2013; Baert *et al*., 2015) and comprises three modules (this platform can be connected to chemostat or segregostat as indicated in Fig. 3): (i) a conventional culture device (Biostat B‐Twin, Sartorius, 2 l), (ii) a physical interface for sampling and dilution comprising peristaltic pumps and mixing chamber, (iii) a detection device, i.e. in our case an Accuri C6 flow cytometer (BD Accuri, San Jose CA, USA). Either module b or c is operated via custom made C++ script.

In short, sample processing comprises the following steps: (i) sample acquisition and online staining, (ii) online FC analysis, (iii) dilution threshold and (iv) feedback control loop.

The sample is fed and removed from the mixing chamber based on silicone tubing (internal diameter: 0.5 mm; external diameter: 1.6 mm, VWR, Belgium) and five peristaltic pumps (400FD/A1 OEM‐pump ~13 rpm and 290 rpm, Watson Marlow). Before and after each experiment, all the connection parts (tubing, pumps and mixed chamber) are continuously cleaned with ethanol and rinsed with filtered PBS.

### Segregostat – Cultivation and sampling

For segregostat cultivations, the medium was continuously fed with salt basal minimal medium except the carbon source (glucose) at a dilution rate of 0.1 h^−1^. The pulse of glucose was fed in the culture medium according to the regulation sequence controlled by the online software, as it will be discussed further. Samples were taken at an interval of 12 min according to the set of dilution sequences that are controlled via the online software. The latter is working through the following sequence of steps. Firstly, the sampling tube was automatically purged for 1 min to eliminate the previous sample and to ensure that fresh sample was collected. At the same time, the mixing chamber and tubing were washed continuously using filtered PBS in order to avoid cross‐contaminations. The next step was to feed the mixed chamber with 500 μl of PI diluted in filtered PBS at a concentration of 4 mg l^−1^ and adjust the given dilution sequence. Then, the sample was mixed with PI followed by incubation at room temperature for 1 min (for *E. coli*) and 3 min (for *P. putida*) strains. Finally, the sample is automatically transferred to C6 FC (BD Accuri C6, BD Biosciences) and is analysed at a medium flow rate (33 μl min^−1^) with a threshold FSC‐H set at 16 000 and 80 000 for *P. putida* and *E. coli* strain respectively. All the data related to the different parameters (mean, median, CV) are available to be displayed in real time during the cultivation.

During sample acquisition, the number of events per microlitre was maintained in the range 500–1500 via a tailor‐made MATLAB script to further avoid doublet detection. Indeed, if the number of cells per microlitre corresponding to the current sample was below 2500 events/s, the actual dilution rate will be maintained for the next sample. Otherwise, the dilution rate was increased or reduced by a factor of 2 if the number of cells per microlitre was above the upper threshold or under the lower threshold respectively.

A tailor‐made MATLAB script based on FC data controlled the activation of the feedback control loop. Thus, cells were gated based on forward scatter (FSC) and red fluorescence (FL3) channel and expressed as a percentage. OM‐permeabilized cells were gated based on a lower threshold on FL3 (fixed as the background fluorescence with non‐stained cells) and an upper threshold (defined as the minimum detectable signal for dead cells). The fraction of OM‐permeabilized cells was kept at 10% by glucose (w/v) pulsing (0.3 g of glucose) with a digital control system comprising a peristaltic pump (Watson Marlow, 101 UR).

### Staining control experiments

For the preparation of non‐viable cells to be used as a positive control for PI staining and flow cytometry gating, 1 ml of cell suspension was heated at 80°C for 1 h. The cells were then washed and resuspended in filtered PBS. Then, 5 μl of propidium iodide (1 mg ml^−1^) was added to the cell suspension and then incubated for 10 min at room temperature. The red fluorescence signal was measured by FC using the parameters described above. Double‐staining experiments with PI/RSG and PI/2‐NBDG were performed for a better characterization of the metabolic properties of OM‐permeabilized cells (Appendix S2).

### Poisson process simulation

Control of population diversification in segregostat was made based on glucose pulsing, where periods with glucose pulses correspond to active phenotypic diversification. In order to determine whether this phenotypic diversification process followed a Poisson processes, several stochastic simulations have been carried out. The first set of simulations was carried out for determining the time required for adding a given amount of glucose pulses if it is assumed that the pulsing dynamics follow a Poisson process with a given glucose pulse addition rate λ (h^−1^). Both the pulse addition rate λ and the number of pulses *n* have been determined experimentally from segregostat experiments. Based on these data, the simulated time for adding n glucose pulses was computed as :

(1) $$t_{n}=\sum_{1}^{n}-\ln\frac{\mathrm{rand}(n)}{\lambda }$$

with rand being a random number generated from a uniform distribution.

The output of the simulations, i.e. the distribution of time required for adding n glucose pulses following Eq. (1), was used for computing the mean of the distribution (denoted as mean time, Fig. 5A).

The second set of simulations was made based on the same algorithm, but the output data were processed for computing the time between two consecutive glucose pulses during segregostat experiments (denoted as waiting time, Fig. 5B). All computations were made based on MATLAB (R2014b) and R.

## Conflict of interests

None declared.

## Supporting information


**Appendix S1.** Strain information.
**Appendix S2**. Dual‐staining flow cytometry experiments.
**Appendix S3**. Dissolved oxygen profiles for segregostat cutlivations.

## References

[mbt213442-bib-0001] Ackermann, M. (2015) A functional perspective on phenotypic heterogeneity in microorganisms. Nat Rev Microbiol 13: 497–508. 26145732 10.1038/nrmicro3491

[mbt213442-bib-0002] Ackermann, M. , and Schreiber, F. (2015) A growing focus on bacterial individuality. Environ Microbiol 17: 2193–2195. 25973653 10.1111/1462-2920.12877

[mbt213442-bib-0003] Baba, T. , Ara, T. , Hasegawa, M. , Takai, Y. , Okumura, Y. , Baba, M. , *et al* (2006) Construction of *Escherichia coli* K‐12 in‐frame, single‐gene knockout mutants: the Keio collection. Mol Syst Biol 2: 0008. 16738554 10.1038/msb4100050 PMC1681482

[mbt213442-bib-0004] Baert, J. , Kinet, R. , Brognaux, A. , Delepierre, A. , Telek, S. , Sorensen, S.J. , *et al* (2015) Phenotypic variability in bioprocessing conditions can be tracked on the basis of on‐line flow cytometry and fits to a scaling law. Biotechnol J 10: 1316–1325. 26179479 10.1002/biot.201400537

[mbt213442-bib-0005] Benzinger, D. , and Khammash, M. (2018) Pulsatile inputs achieve tunable attenuation of gene expression variability and graded multi‐gene regulation. Nat Commun 9: 3521. 30166548 10.1038/s41467-018-05882-2 PMC6117348

[mbt213442-bib-0006] Binder, D. , Drepper, T. , Jaeger, K.‐E. , Delvigne, F. , Wiechert, W. , Kohlheyer, D. , *et al* (2017) Homogenizing bacterial cell factories: analysis and engineering of phenotypic heterogeneity. Metab Eng 42: 145–156. 28645641 10.1016/j.ymben.2017.06.009

[mbt213442-bib-0007] Blake, WJ , KAErn, M , Cantor, CR and Collins, JJ. (2003) Noise in eukaryotic gene expression. Nature 422: 633–637. 12687005 10.1038/nature01546

[mbt213442-bib-0008] Bot, C.T. , and Prodan, C. (2010) Quantifying the membrane potential during *E. coli* growth stages. Biophys Chem 146: 133–137. 20031298 10.1016/j.bpc.2009.11.005

[mbt213442-bib-0009] Briat, C. , and Khammash, M. (2018) Perfect adaptation and optimal equilibrium productivity in a simple microbial biofuel metabolic pathway using dynamic integral control. ACS Synth Biol 7: 419–431. 29343065 10.1021/acssynbio.7b00188

[mbt213442-bib-0010] Briat, C. , Gupta, A. , and Khammash, M. (2018) Antithetic proportional‐integral feedback for reduced variance and improved control performance of stochastic reaction networks. J R Soc Interface 15. pii: 20180079. 10.1098/rsif.2018.0079 PMC6030643 29899158

[mbt213442-bib-0011] Brognaux, A. , Han, S. , Sorensen, S.J. , Lebeau, F. , Thonart, P. and Delvigne, F. (2013) A low‐cost, multiplexable, automated flow cytometry procedure for the characterization of microbial stress dynamics in bioreactors. Microb Cell Factories 12: 100. 10.1186/1475-2859-12-100 PMC4228430 24176169

[mbt213442-bib-0012] del Castillo, T. , Ramos, J.L. , Rodriguez‐Herva, J.J. , Fuhrer, T. , Sauer, U. , and Duque, E. (2007) Convergent peripheral pathways catalyze initial glucose catabolism in *Pseudomonas putida:* genomic and flux analysis. J Bacteriol 189: 5142–5152. 17483213 10.1128/JB.00203-07 PMC1951859

[mbt213442-bib-0013] Chong, Z.‐S. , Woo, W.‐F. , and Chng, S.‐S. (2015) Osmoporin OmpC forms a complex with MlaA to maintain outer membrane lipid asymmetry in *Escherichia coli* . Mol Microbiol 98: 1133–1146. 26314242 10.1111/mmi.13202

[mbt213442-bib-0014] Delvigne, F. , Pecheux, H. , and Tarayre, C. (2015) Fluorescent reporter libraries as useful tools for optimizing microbial cell factories: a review of the current methods and applications. Front Bioeng Biotechnol 3: 147. 26442261 10.3389/fbioe.2015.00147 PMC4585110

[mbt213442-bib-0015] Dusny, C. , Fritzsch, F.S.O. , Frick, O. , and Schmid, A. (2012) Isolated microbial single cells and resulting micropopulations grow faster in controlled environments. Appl Environ Microbiol 78: 7132–7136. 22820335 10.1128/AEM.01624-12 PMC3457477

[mbt213442-bib-0016] Eldar, A. , and Elowitz, M.B. (2010) Functional roles for noise in genetic circuits. Nature 467: 167–173. 20829787 10.1038/nature09326 PMC4100692

[mbt213442-bib-0017] Ferenci, T. (1996) Adaptation to life at micromolar nutrient levels : the regulation of *Escherichia coli* glucose transport by endoinduction and cAMP. FEMS Microbiol Rev 18: 301–317. 8703508 10.1111/j.1574-6976.1996.tb00246.x

[mbt213442-bib-0018] Ferenci, T. (2001) Hungry bacteria ‐ definition and properties of a nutritional state. Environ Microbiol 3: 605–611. 11722540 10.1046/j.1462-2920.2001.00238.x

[mbt213442-bib-0019] Gosset, G. (2005) Improvement of *Escherichia coli* production strains by modification of the phosphoenolpyruvate:sugar phosphotransferase system. Microb Cell Factories 4: 14. 10.1186/1475-2859-4-14 PMC1156936 15904518

[mbt213442-bib-0020] Grunberger, A. , van Ooyen, J. , Paczia, N. , Rohe, P. , Schiendzielorz, G. , Eggeling, L. , *et al* (2013) Beyond growth rate 0.6: *Corynebacterium glutamicum* cultivated in highly diluted environments. Biotechnol Bioeng 110: 220–228. 22890752 10.1002/bit.24616

[mbt213442-bib-0021] Hancock, R.E.W. , and Brinkman, F.S.L. (2002) Function of *Pseudomonas porins* in uptake and efflux. Annu Rev Microbiol 56: 17–38. 12142471 10.1146/annurev.micro.56.012302.160310

[mbt213442-bib-0022] van Heerden, J.H. , Kempe, H. , Doerr, A. , Maarleveld, T. , Nordholt, N. , and Bruggeman, F.J. (2017) Statistics and simulation of growth of single bacterial cells: illustrations with *B. subtilis* and *E. coli* . Sci Rep 7: 16094. 29170466 10.1038/s41598-017-15895-4 PMC5700928

[mbt213442-bib-0023] Kaern, M. , Elston, T.C. , Blake, W.J. , and Collins, J.J. (2005) Stochasticity in gene expression: from theories to phenotypes. Nat Rev Genet 6: 451–464. 15883588 10.1038/nrg1615

[mbt213442-bib-0024] Kiviet, D.J. , Nghe, P. , Walker, N. , Boulineau, S. , Sunderlikova, V. , and Tans, S.J. (2014) Stochasticity of metabolism and growth at the single‐cell level. Nature 514: 376–379. 25186725 10.1038/nature13582

[mbt213442-bib-0025] Kleijn, I.T. , Krah, L.H.J. , and Hermsen, R. (2018) Noise propagation in an integrated model of bacterial gene expression and growth. PLoS Comput Biol 14: e1006386. 30289879 10.1371/journal.pcbi.1006386 PMC6192656

[mbt213442-bib-0026] Levine, J.H. , Lin, Y. , and Elowitz, M.B. (2013) Functional roles of pulsing in genetic circuits. Science 342: 1193–1200. 24311681 10.1126/science.1239999 PMC4100686

[mbt213442-bib-0027] Liu, X. (1998) Regulation of porin‐mediated outer membrane permeability by nutrient limitation in Escherichia coli. J Bacteriol 180: 3917–3922. 9683489 10.1128/jb.180.15.3917-3922.1998 PMC107376

[mbt213442-bib-0028] Marquardt, D. , Geier, B. , and Pabst, G. (2015) Asymmetric lipid membranes: towards more realistic model systems. Membranes 5: 180–196. 25955841 10.3390/membranes5020180 PMC4496639

[mbt213442-bib-0029] Milias‐Argeitis, A. , Rullan, M. , Aoki, S.K. , Buchmann, P. , and Khammash, M. (2016) Automated optogenetic feedback control for precise and robust regulation of gene expression and cell growth. Nat Commun 7: 12546. 27562138 10.1038/ncomms12546 PMC5007438

[mbt213442-bib-0030] Nikel, P.I. , Martinez‐Garcia, E. , and de Lorenzo, V. (2014) Biotechnological domestication of pseudomonads using synthetic biology. Nat Rev Microbiol 12: 368–379. 24736795 10.1038/nrmicro3253

[mbt213442-bib-0031] Nikolic, N. , Barner, T. , and Ackermann, M. (2013) Analysis of fluorescent reporters indicates heterogeneity in glucose uptake and utilization in clonal bacterial populations. BMC Microbiol 13: 258. 24238347 10.1186/1471-2180-13-258 PMC3840653

[mbt213442-bib-0032] Patange, O. , Schwall, C. , Jones, M. , Villava, C. , Griffith, D.A. , Phillips, A. , *et al* (2018) Escherichia coli can survive stress by noisy growth modulation. Nat Commun 9: 5333. 30559445 10.1038/s41467-018-07702-z PMC6297224

[mbt213442-bib-0033] Shi, L. , Gunther, S. , Hubschmann, T. , Wick, L.Y. , Harms, H. , and Muller, S. (2007) Limits of propidium iodide as a cell viability indicator for environmental bacteria. Cytom Part J Int Soc Anal Cytol 71: 592–598. 10.1002/cyto.a.20402 17421025

[mbt213442-bib-0034] Wides, A. and Milo, R. Understanding the dynamics and optimizing the performance of chemostat selection experiments. arXiv:180600272.

[mbt213442-bib-0035] Wortel, M.T. , Bosdriesz, E. , Teusink, B. , and Bruggeman, F.J. (2016) Evolutionary pressures on microbial metabolic strategies in the chemostat. Sci Rep 6: 29503. 27381431 10.1038/srep29503 PMC4933952

[mbt213442-bib-0036] Yeow, J. , Tan, K.W. , Holdbrook, D.A. , Chong, Z.‐S. , Marzinek, J.K. , Bond, P.J. , *et al* (2018) The architecture of the OmpC‐MlaA complex sheds light on the maintenance of outer membrane lipid asymmetry in *Escherichia coli* . J Biol Chem 293: 11325–11340. 29848551 10.1074/jbc.RA118.002441 PMC6065193

