# Supplementary material for: Segregostat: a novel concept to control phenotypic diversification dynamics on the example of Gram‐negative bacteria
Source: Microb Biotechnol. 2019 May 29;12(5):1064–75. doi: 10.1111/1751-7915.13442 (PMC6680609; doi:10.1111/1751-7915.13442)
Supplement: Supplementary file 1 — Appendix S1. Strain information. Appendix S2. Dual‐staining flow cytometry experiments. Appendix S3. Dissolved oxygen profiles for segregostat cutlivations. [file MBT2-12-1064-s001.pdf]

Supplementary material

Hosni Sassi[1*], Thai Minh Nguyen[1*], Samuel Telek[1], Guillermo Gosset[2], Alexander Grünberger[3], Frank Delvigne[1]

[1] Terra research and teaching centre, Microbial Processes and Interactions (MiPI), Gembloux Agro-Bio Tech, University of Liège, Gembloux, Belgium.

[2] Departamento de Ingeniería Celular y Biocatálisis, Instituto de Biotecnología, Universidad Nacional Autónoma de México, Cuernavaca, Morelos, México.

[3]Multiscale Bioengineering, Bielefeld University, Universitätsstraße 25, 33615 Bielefeld, Germany

Segregostat: A novel concept to control phenotypic diversification dynamics on the example of Gram-negative bacteria

Appendix S1 – Strain information

E. coli JW2203-1 Δ*ompC* have been selected based on a prescreening test since it was able to display a higher diversification ratio by comparison with wild type and other porin mutants (i.e. Δ*ompF* and Δ*lamB*). All these strains (table S1) have been stained with PI and analyzed by FC (Figure S1).

Cultures were performed in microplates in Bioscreen C at 37°C on a defined mineral salt medium containing (in g/L): K_2HPO_4 14.6, $NaH_2PO_4.2H_2O$ 3.6, Na_2SO_4 2, $(NH_4)_2SO_4$ 2.47, NH_4Cl 0.5, $(NH_4)2$-H-citrate 1, glucose 5, thiamine 0.01, kanamycin 0.1. Thiamine and kanamycin were sterilised by filtration (0.2µm). The medium is supplemented with 3 mL/L trace solution, 3 mL/L $FeCl_3.6H_2O$ solution (16.7 g/L), 3 mL/L EDTA solution (20.1 g/L) and 2 ml/L $MgSO_4$ solution (120 g/L). The trace solution contains (in g/L): $CoCl_2.H_2O$ 0.74, $ZnSO_4.7H_2O$ 0.18, $MnSO_4.H_2O$ 0.1, $CuSO_4.5H_2O$ 0.1 and $CoSO_4.7H_2O$ 0.21.

Table S1: list of strains

**Name**	**Genotype**	CGSC#
**<u>BW25113</u> WT**	F-, *Δ(araD-araB)567*, *ΔlacZ4787*(::rrnB-3), λ^-, *rph-1*, *Δ(rhaD-rhaB)568*, *hsdR514*	7636
**<u>JW3996-1</u>** ***ΔlamB***	F-, *Δ(araD-araB)567*, *ΔlacZ4787*(::rrnB-3), λ^-, *rph-1*, *Δ(rhaD-rhaB)568*, *ΔlamB732::kan*, *hsdR514*	10877
**<u>JW0912-1</u>** ***ΔompF***	F-, *Δ(araD-araB)567*, *ΔlacZ4787*(::rrnB-3), λ^-, *ΔompF746::kan*, *rph-1*, *Δ(rhaD-rhaB)568*, *hsdR514*	8925
**<u>JW2203-1</u>** ***ΔompC***	F-, *Δ(araD-araB)567*, *ΔlacZ4787*(::rrnB-3), λ^-, *ΔompC768::kan*, *rph-1*, *Δ(rhaD-rhaB)568*, *hsdR514*	9781

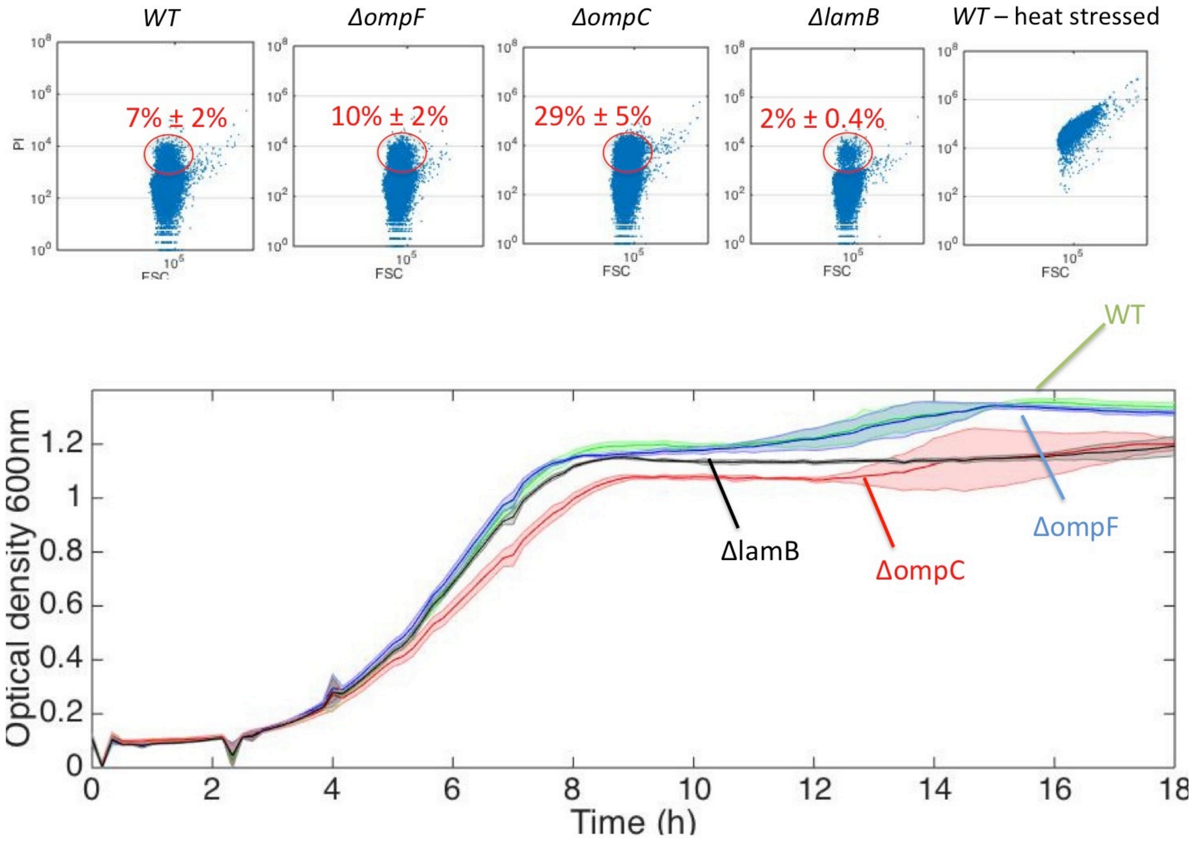

Figure S1 : (TOP) permeabilization of OM of E. coli leads to the accumulation of PI in the perisplasmic space. Flow cytometry analyses (x-axis : FSC, i.e. forward scatter proportional to cell size ; y-axis : PI level, i.e. fluorescence related to PI accumulation in cells) of wild type (WT), deletion mutants and wild type exposed to heat stress (60°C for 30 minutes). (BOTTOM) Evolution of optical density in Bioscreen C microplate cultivation device (cultures have been made in triplicates, mean and standard deviation are indicated).

Samples have been taken after 18h of cultivation in Bioscreen C, stained with PI and analyzed by FC.

Appendix S2

For further characterizing the metabolic features behind OM-permeabilized cells (i.e. partially staining with propidium iodide/PI), PI staining has been combined with complementary fluorescent probes, i.e. redox green sensor (RSG) and 2-[N-(7-nitrobenz-2-oxa-1,3-diazol-4-yl)amino]-2-deoxy-D-glucose (2-NBDG). PI, RSG and 2-NBDG were purchased from Thermo Fisher Scientific (Belgium). The dyes stock solutions were prepared as following: PI (1, 49 mM solution in sterile water), RSG (1 mM solution in sterile water) and 2NBDG (100 µM solution in sterile water). Briefly, cells were grown in flask in the defined medium supplemented with 5 g/L of glucose as described above. Cells were harvested at the end and at the mid-exponential phase of *E. coli W3110* and *Pseudomonas putida KT2440* strain respectively. The cells were diluted in 1 ml filtered PBS buffer to about 10^7 cells/ml and the dyes were then added at the appropriate final concentrations. *E. coli* and *P. putida* cells were stained with five or one µl of PI solution respectively, or in mixture with one µl of RSG (for dual staining experiments). For the assessment of glucose uptake, cells were stained with PI in mixture with 2-NBDG at a final concentration of 10 µM and 90 µM for *E. coli* and *P. putida* strain respectively. The samples were then mixed and incubated in the dark for 1 hour at room temperature. In order to avoid spectral overlap, samples have been analyzed at two separate excitation wavelengths for PI and RSG/2-NBDG respectively. The sample acquisition was performed using the Attune™ NxT Flow Cytometer (Life Technologies TM) instrument at 200 µl/minute flow rate. The RSG and 2 NBDG fluorescence intensities of stained cells were recovered in the BL1 channel (excitation 488 nm, emission filter 530/30 nm). The PI fluorescence was recovered in the YL2 (excitation

561 nm, emission filter 610/20 nm). For each sample, 40,000 events were recorded and data analysis was performed using FlowJo software.

For both model systems, cells exhibited fluorescence associated with RSG and 2-NBDG uptake, suggesting that these cells are still metabolically active (Figure S2).

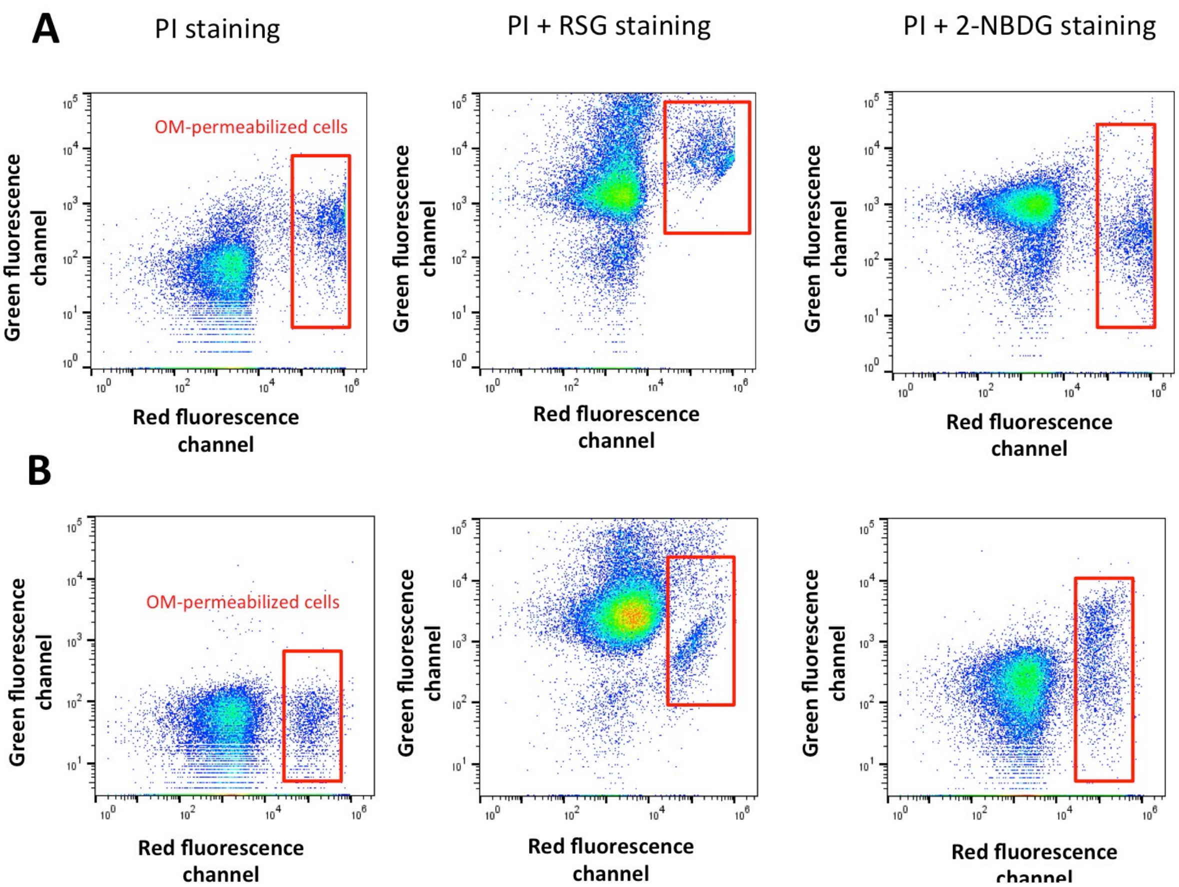

Figure S2.1: flow cytometry analysis of **A** : *E. coli* and **B**: *P. putida* upon staining with PI and double staining with either PI/RSG or PI/2-NBDG. Each analysis has been performed in triplicate and only a representative flow cytometry profile is shown.

Appendix S3

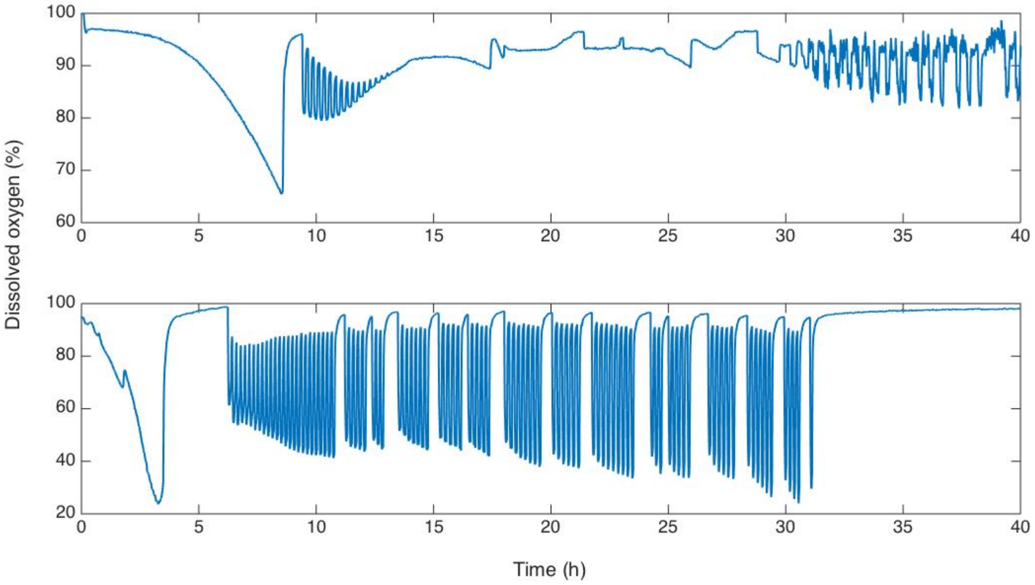

Figure S3: dissolved oxygen concentration profiles recorder during segregostat cultivations (see Figure 4A for 4B in the main manuscript for the corresponding diversification profiles. TOP : *E. coli* cultivation. BOTTOM : *P. putida* cultivation.
